# Religious Coping Amidst a Pandemic: Impact on COVID-19-Related Anxiety

**DOI:** 10.1007/s10943-021-01385-5

**Published:** 2021-08-18

**Authors:** Tommy DeRossett, Donna J. LaVoie, Destiny Brooks

**Affiliations:** Department of Psychology, Saint Louis University, St. Louis, MO 63108 USA

**Keywords:** Religious coping, COVID-19, Health, Religion, USA

## Abstract

Religious coping is one potential strategy to manage stressors. Positive religious coping has been linked to better physical and mental health outcomes, while negative religious coping has been associated with increased stress and anxiety. The primary objective of this study was to examine individuals’ use of religious coping during the COVID-19 pandemic. We examined the relationship between COVID-19 anxiety and religious coping in a national sample of 970 individuals located within the USA recruited via Amazon’s Mechanical Turk (MTurk) between September 12, 2020, and September 25, 2020. Findings indicate negative religious coping is most strongly associated with COVID-19 anxiety, as higher levels of negative religious coping were positively related to COVID-19 anxiety. In a moderated multiple regression wherein positive religious coping and negative religious coping were included in an interaction term, only negative religious coping was significantly associated with COVID-19 anxiety. This may have been due, in part, because individual’s typical religious engagement was disrupted by social distancing and isolation measures. When accounting for participant age, sex, religious beliefs and behaviors, and negative religious coping, positive religious coping was negatively, although weakly, associated with COVID-19 anxiety. These findings suggest that negative religious coping has a stronger association with COVID-19 anxiety than positive religious coping.

## Introduction

Near the end of 2019, COVID-19 was identified by the World Health Organization and within months was rapidly spreading globally. To attempt to contain the spread of the virus, the USA enacted a number of measures, including mask wearing, social distancing, and even shelter at home, or quarantine, orders in many states and municipalities. While these measures were enacted to control physical spread of the virus, such measures can do significant psychological harm, negatively impacting people’s reported levels of stress and anxiety (Huremović, 2019). Indeed, a survey conducted by the American Psychiatric Association (APA) early in the US pandemic response noted that 36% of Americans reported that the COVID-19 was having a serious impact on their mental health, with most respondents (59%) also reporting the COVID-19 as having significant negative impact on their day-to-day lives (American Psychiatric Association, 2020a). Six months later a second survey (APA, 2020b) found that 62% of respondents indicated feeling more anxious than they had at the same time last year, up from 36% averaged over the previous three years, with 75% of respondents listing COVID-19 as a top concern. These findings are consistent with a review by Brooks et al. (2020) reporting that the consequences of the types of quarantine measures like those enacted for the COVID-19 pandemic can have devastating effects on mental health, including exhaustion, isolation, boredom, frustration, and symptoms of post-traumatic stress.

The pervasiveness of religiosity has provided the opportunity for religious leaders to act as agents of change for better or worse during the COVID-19 pandemic. Religious communities have been called upon to work with secular and political organizations to promote health (Williams et al., 2021). However, medicinal and cultural competence of the religious leaders is an important variable when assessing their role to guide individuals in times of crisis. For example, in Africa individuals report that their religious leaders may influence them to avoid seeking treatment for physical or mental health issues by claiming that their ills are a form of “payback” for wrongdoings from one’s past (Nyashanu et al., 2021). In Iran, Clerics have served as important community organizers during the COVID-19 pandemic (Lebni et al., 2021). However, Lebni et al. (2021) found that some leaders opposed vaccination and isolation efforts which may have led to increased infection rates. Because of the power of their suggestions, it is important for religious leaders to recognize their power and make well-educated statements for the betterment of society.

## Religious Coping

Many people utilize religion as a method of mental or physical improvement as religiosity has been linked to higher levels of mental and physical health when incorporated into treatment plans (Koenig, 2012; Wang et al., 2003). The potential for religion as a coping strategy has been long discussed (Pargament, 1997). According to Pargament (1997), religious coping, while generally related to ideas about the sacred, encompasses a wide range of efforts informed by one’s religion to cope with life stressors. These include behaviors such as prayer, confession, seeking spiritual support from clergy or others, and acceptance of circumstances as representing the will of God. Religious coping may be beneficial or costly depending on whether one engages in positive or negative coping methods (Pargament et al., 2000).

Positive religious coping (PRC) relies on a secure relationship with God/the divine, belief that life is meaningful, and spiritual connectedness to others as means to alleviate negative consequences of life stressors. Negative religious coping (NRC), however, reflects conflict and struggle with the divine and others, as well as a struggle to find significance in life. PRC is generally associated with favorable psychological outcomes, adaptation, and resiliency, while NRC is generally associated with psychological distress and poorer mental health (Koenig, 2018; Pargament et al., 2011).

There is no doubt that the COVID-19 pandemic represents a significant stressor to individuals worldwide, as indicated by recent studies (e.g., Lei et al., 2020; Xiong et al., 2020) that show increases in symptoms of depression and anxiety relative to pre-COVID-19 years. There is an indication that individuals have increasingly turned to religion since the beginning of the pandemic, and it has even been a positive indicator of positive affect (Zacher & Rudolph, 2021). Bentzen (2020) reports that Google searches for the word “prayer” have risen to the highest levels ever recorded for that search term. Lucchetti et al., (2020) note an increase in the general use of religious and spiritual practices to alleviate the negative consequences of social isolation measures in the pandemic. Investigating religious coping specifically, Thomas and Barbato (2020) found an inverse relationship between positive religious coping and general mental health early in the pandemic in a sample of Muslims residing in the United Arab Emirates. Similar findings of positive religious coping on stress levels during the pandemic among American Orthodox Jews have been reported by Pirutinsky et al. (2020).

While these findings are notable regarding the impact of religious coping on general levels of anxiety and psychological distress during the pandemic, neither specifically address how religious coping may be used to alleviate or exacerbate COVID-19-specific related anxiety and distress. Lee (2020) has recently validated a brief mental health screener, the COVID-19 Anxiety Scale (CAS), that is designed to be used for detection of anxiety specifically related to COVID-19, a form of anxiety that some have labeled Coronaphobia (Asmundson & Taylor, 2020a) or COVID Stress Syndrome (Asmundson & Taylor, 2020b). Individuals with Coronaphobia not only show elevated levels of depression and generalized anxiety, but also significant increases in death anxiety (Lee et al., 2020). Missing from these studies is an analysis of what types of coping strategies people employ to alleviate their COVID-19-related psychological distress.

## Current Study

The current study specifically examines this relationship, by administering the CAS (Lee, 2020) and the Brief RCOPE (Pargament et al., 2011) to a sample of individuals across the USA. Although the nature of this project was largely exploratory, we did specifically hypothesize an inverse relationship between positive religious coping (PRC) and reported levels of COVID-19 anxiety (Cov-Anx), as well as a positive association between reported levels of negative religious coping (NRC) and reported levels of Cov-Anx. Because heightened religiosity has been reported to be associated with greater use of positive religious coping methods (Pargament et al., 2011), we included a measure of religious behavior/religious experience (i.e., religiosity) in our study, the Religious Beliefs and Behaviors Questionnaire (RBBQ; Connors et al., 1996), to determine whether religious behavior in general is associated with Cov-Anx.

## Method

### Participants

Participants in this study were recruited through Amazon’s Mechanical Turk (MTurk) to complete an online survey. Data collection took place between September 12, 2020, and September 25, 2020. To qualify for the study, participants were required to be 18 years of age or older, a resident of the USA, and to have an MTurk approval rating of 95% or higher. Participants who completed the survey were compensated $0.50 for their participation. Throughout the survey attention checks were included to improve the quality of responses. Additionally, a unique code was provided to participants upon survey completion to ensure their attention and full participation. If the participants provided an incorrect code, or failed to complete the study, their data were removed from the sample.

The original sample consisted of 1,062 participants. After the removal of individuals who failed to complete the study, failed attention checks, or did not follow instructions, 970 participants remained. Participants ranged from 20 to 79 years of age (*M* = 38.43, *SD* = 11.28) with two participants choosing not to share their age. The participants mostly identified as male (*n* = 546, or 56%; female *n* = 418, or 43%), two identifying as transgender, one identifying as nonbinary, and three choosing not to share their gender identity. The participants mostly identified as Caucasian (*n* = 555; 57%), with 204 (21%) participants identifying as African-American, 48 (5%) identifying as Asian, three (< 1%) identifying as Pacific Islander, 52 (5%) identifying as Hispanic, six identifying as bi-racial, 71 (7%) identifying as Native American, and 29 choosing not to disclose their ethnic identity. Finally, regarding religious identity, 435 participants identified as religious (45%), 221 identified as spiritual (23%), 154 identified as agnostic (16%), 82 identified as unsure (9%), 76 identified as atheist (16%), and two participants declined to report their religious identity.

### Measures

Participants were asked to complete four measures: (1) the COVID-19 Anxiety Scale; (2) The Brief Religious Coping Inventory (Brief RCOPE); (3) the Religious Background and Behaviors Questionnaire (RBBQ); and (4) a brief demographics questionnaire.

The *COVID-19 Anxiety Scale* (*CAS*; Lee, 2020) is a 5-item instrument assessing five distinct physiological symptoms of anxiety and fear related to COVID-19, including dizziness, sleep disturbances, tonic immobility, appetite loss, and abdominal distress (see Table 1). Overall, this measure has shown acceptable levels of sensitivity (90%) and specificity (85%) as a mental health screener, as well as high construct validity (Lee, 2020). Within our sample, the scale showed strong levels of reliability (α = 0.94).

**Table 1:** The Coronavirus Anxiety Scale (CAS; Lee, 2020) items and anchors

How often have you experienced the following activities over **the last 2 weeks?**				
Not at all (0)	Rare, less than a day or two (1)	Several days (2)	More than 7 days (3)	Nearly every day over the last 2 weeks (4)
I felt dizzy, lightheaded, or faint, when I read or listened to news about the coronavirus				
I had trouble falling or staying asleep because I was thinking about the coronavirus				
I felt paralyzed or frozen when I thought about or was exposed to information about the coronavirus				
I lost interest in eating when I thought about or was exposed to information about the coronavirus				
I felt nauseous or had stomach problems when I thought about or was exposed to information about the coronavirus				

The *Brief Religious Coping Inventory* (*Brief RCOPE*; Pargament et al., 2011) consists of 14 items divided into two subscales: positive (α = 0.90) and negative religious coping (α = 0.93). The scale assesses the degree to which participants engage in both positive (e.g., *looked for stronger connection with God*) and negative religious coping (e.g., *felt punished by God for my lack of devotion*) using a Likert-type scale ranging from 1 (*not at all*) to 4 (*a great deal*). We modified the usual instructions, asking participants to specifically consider the extent to which they had engaged in the behaviors described in the items since the start of the pandemic.

The *Religious Background and Behavior Questionnaire (RBBQ;* Connors et al., 1996*)* is a 13-item scale designed to measure religious practices, e.g., the use of prayer and meditation, scripture reading, and attendance at worship services. The first section (one item) asks participants to select their religious identity from a list of six potential options: Atheist, Agnostic, Unsure, Spiritual, or Religious. Each option is defined for the participant, and they are asked to select the item which best describes them. As an example, Spiritual is defined as *I believe in God or a Higher Power, but I’m not religious.*

The second section (6 items) measures the frequency of engagement (RBBQ-Freq; α = 0.90) in or formal practice of religious behavior over the past year using an 8-point Likert scale, ranging from 1 (*never*) to 8 (*more than once a day*). The final section is made up of 6 items assessing a participant’s overall experience (RBBQ-Exp; α = 0.75) with religious behaviors (a component labeled *God Consciousness*), intended to capture behaviors that are typically associated with religiosity (e.g., *attended worship services regularly*). Participants are asked to respond on a 3-point scale to items (1 = *never;* 2 = *yes, in the past but not now;* 3 = *yes, and I still do*). Finally, participants were asked to provide demographic information. This included: age, gender identity, ethnicity, education level, state of residence, and whether or not they, a close friend, or a relative had been diagnosed with the COVID-19 (Table 2).

**Table 2:** Demographic information collapsed across national region

	Midwest	Northeast	Pacific	South	West
Age	38.22 (*11.40*)	39.87 (*11.98*)	42.67 (*21.94*)	37.71 (*10.86*)	38.85 (*11.46*)
COVID-19 diagnosis (yes)	41.73%	40.12%	0.00%	48.49%	50.19%
*Gender*					
Male	86	91	2	172	153
Female	49	76	1	160	111
*Ethnicity*					
White	77	102	1	201	144
Non-white	57	59	2	124	112
*Education level*					
College	132	164	2	316	259
No college	7	3	1	15	4

Standard deviations appear in parentheses for means in age row. Percentages for COVID-19 diagnosis represent the percentage of participants who selected “yes” when asked if they, a close friend, or relative had been diagnosed with COVID-19. States were assigned to regions per the US Census Bureau’s records. Midwest = ND, SD, NE, KS, MO, IA, MN, WI, IL, IN, MI, OH. Northeast = ME, NH, VT, MA, CT, RI, NJ, PA, NY. Pacific = HI, AL. South = DE, MD, WV, VA, NC, SC, GA, FL, AL, TN, KY, WV, MS, AR, LA, TX, OK. West = WA, OR, CA, NV, AZ, NM, CO, UT, WY, ID, MT

## Data Analysis

All analyses were conducted within the RStudio statistical computing environment (RStudio, 2020). Each analysis was conducted with an alpha value of *p* < 0.05. Our data analysis plan involved multiple steps. First, we planned to conduct a Pearson’s correlation analysis to determine whether the expected relationships between PRC, NRC, and Cov-Anx would be supported. The correlation was also designed to include potential covariates including age, sex, COVID-19 diagnosis, and religious activities. Then, to better understand the underlying relationships, a series of multiple and moderated regressions were planned. Additionally, an ANOVA was planned to analyze potential differences in levels of religious coping and COVID-19 anxiety per religious identity. Finally, a series of t-tests were planned to analyze potential differences in COVID-19 anxiety, religious coping, or religious activities between individuals with or without a COVID-19 diagnosis for themselves, a close friend, or a family member.

## Results

### Analysis Overview

Prior to testing our specific research hypotheses about the relationship between religious coping and COVID-19-related anxiety, we calculated descriptive statistics (e.g., means, standard deviations, and Cronbach’s alphas) in addition to preliminary Pearson’s pairwise correlational analyses to assess the general pattern of results and overall associations amongst variables (see Table 3). A few correlations are worth noting. First, PRC (*r* = 0.39) and NRC (*r* = 0.74) were both positively correlated with reported levels of Cov-Anx. PRC and NRC were also correlated with one another (*r* = 0.59). Second, RBBQ religious experience (RBBQ-Exp) scores were positively correlated with PRC (*r* = 0.58) and NRC (*r* = 0.61). Levels of PRC (*r* = 0.73) and NRC (*r* = 0.61) were also both positively associated with frequency of religious behavior (RBBQ-Freq). Of particular note, there did not seem to be an association between COVID-19 experience and either religious coping or Cov-Anx. However, sex and age were correlated with predictor and outcome variables and were therefore controlled for in all subsequent analyses. These findings suggested a need to further examine the relationship between religious coping, religious behaviors, and Cov-Anx, through a series of multiple regressions, reported below. COVID-19 experience was not included in subsequent analyses as it was not correlated with any other variables.

**Table 3:** Correlations, means, and standard deviations

	PRC	NRC	RBBQ-Exp	RBBQ-Freq	Cov-Anx	Age	Sex	COVID
PRC	−	0.59*	0.58*	0.73*	0.39*	0.02	− 0.12*	− 0.01
NRC		−	0.27*	0.61*	0.74*	− 0.12*	− 0.15*	− 0.03
RBBQ-Exp			−	0.61*	0.12*	0.04	− 0.04	− 0.01
RBBQ-Freq				−	0.50*	0.02	− 0.14*	− 0.02
Cov-Anx					−	− 0.13*	− 0.13*	− 0.01
Age						−	0.03	0.02
Sex							−	0.01
COVID								−
Mean	2.78	2.45	2.43	5.36	1.75	38.43	−	−
Standard Deviation	0.77	0.90	0.44	1.74	1.22	11.28	−	−

^*^p < *.05*. *PRC* Positive religious coping, *NRC* Negative religious coping, *RBBQ-EXP* RBBQ experiences subscale, *RBBQ-Freq* RBBQ Behavior Frequency Subscale, *Cov-Anx* COVID-19 anxiety, sex: male = 1, female = 2; COVID = experience with COVID-19, scored yes/no

### Regression Analyses

Our primary research question involved the relationship between religious coping and Cov-Anx. Because both PRC and NRC were found to be correlated with Cov-Anx, as well as each other, we conducted a multiple regression analysis to isolate the relative contribution of each to Cov-Anx scores when controlling for age and gender. The results of this analysis, as seen in Table 4, indicate that religious coping in general accounts for 54.5% of the variance in Cov-Anx, *F*(4, 929) = 280.4, *p* < 0.001.

**Table 4:** The association of COVID-19 anxiety scores with both positive and negative religious coping scores and their interaction term

Predictor	*β*	SE	*t*-value	Sig. *t*
*Model 1: Simple effects regression*				
(Intercept)	− 0.46	0.16	− 2.12	0.04
PRC	− 0.06*	0.04	− 2.26	0.02
NRC	0.77*	0.04	27.57	0.00
Sex	− 0.02	0.45	− 1.01	0.31
Age	− 0.03	0.00	− 1.25	0.22
*R*^*2*^ = 0.545*				
*Model 2: Simple effects plus interaction*				
(Intercept)	− 0.69	0.26	− 2.68	0.01
PRC	0.01	0.08	0.20	0.85
NRC	0.93*	0.13	9.58	0.00
Sex	− 0.02	0.05	− 0.94	0.35
Age	− 0.03	0.00	− 1.14	0.25
PRC x NRC	− 0.04	0.04	− 1.74	0.08

*R*^*2*^ = 0.546*, ∆ R^*2*^ = *.001, ns*

^*^p < .*05. PRC* positive religious coping, *NRC* negative religious coping, sex: male = 1, female = 2

The results of this analysis also suggest that while both PRC and NRC contribute to the overall fit of the model, the relative contribution of PRC and NRC differ (PRC_*β*_ = -0.06, NRC_*β*_ = 0.77). To elaborate on these findings, we conducted a moderated regression that included the interaction of PRC and NRC in the model to determine whether the interaction term would account for more variance in Cov-Anx. The model was significantly associated with increased levels of Cov-Anx (*F*(5, 928) = 225.4, *p* < 0.001), but the interaction between positive and negative religious coping was only marginally significant (*p* = 0.08). Additionally, the difference in explained variance between the two models was only marginally significant (*F*(1, 931) = 3.38, *p* = 0.07). Therefore, we concluded that the interaction between positive and negative religious coping did not explain any additional variance in levels of Cov-Anx.

We also were interested in general religiosity, as measured by the RBBQ, and its relationship with Cov-Anx. First, the joint effects of the RBBQ-Exp and RBBQ-Freq subscales were analyzed in a simple multiple regression (see Table 5). This analysis revealed a relationship between the RBBQ subscales and Cov-Anx (*F*(4, 933) = 113.10, *p* < 0.001) but indicated that the RBBQ-Exp subscale is negatively associated with Cov-Anx, while RBBQ-Freq is positively associated with Cov-Anx. Because these subscale scores appear to provide differential impact on Cov-Anx, we conducted an additional regression analysis that included the interaction between these variables to determine the additive and moderating associations between religious beliefs, behaviors, and Cov-Anx. This analysis revealed a reliable interaction between the RBBQ frequency and experience subscales, such that the frequency of religious behaviors was associated with Cov-Anx, but overall religious experience was not (*F*(5, 932) = 93.64, *p* < 0.001). The change in variance explained between the two models was statistically significant.

**Table 5:** The association between COVID-19 anxiety scores and the RBBQ

Predictor	*β*	SE	*t*-value	Sig. *t*
*Model 1: Simple effects regression*				
(Intercept)	1.30	0.22	7.93	0.00
RBBQ-Freq	0.67*	0.02	19.68	0.00
RBBQ-Exp	− 0.29*	0.09	− 8.49	0.00
Sex	− 0.05	0.05	− 1.82	0.06
Age	− 0.13*	0.00	− 4.66	0.00
*R*^*2*^ = 0.310*				
*Model 2: Simple effects plus interaction regression*				
(Intercept)	− 0.05	0.45	1.14	0.18
RBBQ-Freq	1.05*	0.09	8.79	0.00
RBBQ-Exp	− 0.07	0.21	− 0.92	0.44
Sex	− 0.05	0.06	− 1.78	0.06
Age	− 0.13*	0.00	− 4.54	0.00
RBBQ-Freq x RBBQ-Exp	− 0.04*	0.04	− 3.32	0.00

*R*^*2*^ = 0.318* ∆ R^*2*^ = .009*

^*^p < *.05. RBBQ-EXP* RBBQ experiences subscale, *RBBQ-Freq* RBBQ Behavior Frequency Subscale, sex: male = 1, female = 2

Overall, these separate analyses of religious coping and religious beliefs and behaviors (i.e., religiosity) suggest a complicated relationship between religion and Cov-Anx, so we conducted a hierarchical multiple regression to examine their joint effects on Cov-Anx (Table 6). Because NRC had the strongest association with Cov-Anx in our previous analyses, it was entered first into the model along with sex and age (*F*(3, 937) = 373.60,* p* < 0.001, *R*^*2*^ = 0.54). Next, we added RBBQ-Freq (*F*(4, 928) = 283.8,* p* < 0.001, *R*^*2*^ = 0.55), then RBBQ-Exp, (*F*(5, 921) = 252.3, *p* < 0.001, *R*^*2*^= 0.58), and finally PRC (*F*(6, 914) = 212.4,* p* < 0.001, *R*^*2*^ = 0.58). Each predictor variable was significant at each step of the hierarchical multiple regression, with the final model revealing positive relationships between NRC and RBBQ-Freq with Cov-Anx, but PRC and RBBQ-Exp being inversely associated with Cov-Anx. Age was significantly associated with Cov-Anx until the inclusion of PRC, with age representing lower levels of Cov-Anx up to that point. These results are consistent with our hypotheses.

**Table 6:** The association between COVID-19 anxiety scores, religious coping, and RBBQ subscales

	Predictor	*β*	SE	*t*-value	Sig. *t*	Fit (R^2^)	Change
Step one	NRC	0.73	0.03	33.48	0.00		
	Sex	− 0.02	0.05	− 0.86	0.40		
	Age	− 0.04	0.00	− 1.64	0.10		
						0.543*	
Step two	NRC	0.67*	0.04	23.72	0.00		
	Sex	− 0.02	0.05	− 0.65	0.51		
	Age	− 0.05*	0.00	− 2.18	0.03		
	RBBQ-Freq	0.10*	0.02	3.61	0.00		
						0.548*	∆ R^2^ =0 .005
Step three	NRC	0.64*	0.04	23.14	0.00		
	Sex	− 0.00	0.04	− 0.16	0.862		
	Age	− 0.04*	0.00	− 2.01	0.04		
	RBBQ-Freq	0.24*	0.02	7.33	0.00		
	RBBQ-Exp	− 0.20*	0.08	− 7.29	0.00		
						0.578*	∆ R^2^ =0 .030*
Step four	NRC	0.67*	0.04	23.69	0.00		
	Sex	− 0.01	0.04	− 0.35	0.73		
	Age	− 0.04	0.00	− 1.67	0.10		
	RBBQ-Freq	0.28*	0.03	7.84	0.00		
	RBBQ-Exp	− 0.17*	0.08	− 5.78	0.00		
	PRC	− 0.11*	0.05	− 3.52	0.00		
						0.580*	∆ R^2^ = 0.002

^*^ *p* < .05. *PRC* Positive religious coping, *NRC* Negative religious coping, *RBBQ-EXP RBBQ* Experiences subscale, RBBQ-Freq RBBQ behavior frequency subscale; Sex: Male = 1, Female = 2. Sex was removed after the first step because it was not associated with COVID-19 anxiety

Additionally, a variance inflation factor (VIF) test was applied to this model as a test of multicollinearity due to the inclusion of several moderate to highly correlated variables. The VIF test was accessed through the “car” package (Fox, 2019) within the RStudio environment. Variance inflation factors are considered an acceptable measure of multicollinearity, with values over 5 suggesting high levels of multicollinearity that demand attention (Daoud, 2017). The VIF values of our measures ranged between 1.03 (participant sex) and 2.80 (RBBQ-Freq) suggesting that multicollinearity was not an issue. Daoud (2017) also suggests that inflated standard errors are an indication of multicollinearity, which was not an issue within our analyses (*SEs* < 0.09). In conclusion, it does not appear that multicollinearity is a concern for the current study.

### Religious Identity and COVID-19 Anxiety

We purposely sampled a religiously diverse sample of participants to determine the role of religious identity in the relationship between religious coping and Cov-Anx. A between-groups ANOVA comparing reported levels of Cov-Anx between the various religious identities (Atheist, Agnostic, Unsure, Spiritual, or Religious) revealed an overall difference in Cov-Anx between groups, *F* (4, 945) = 2.59, *p* = 0.036 (see Table 7). To elucidate this difference, contrasts using Tukey’s HSD test revealed that agnostics reported the highest level of Cov-Anx as compared to each of the other religious identities, with all *p’s* < 0.01.

**Table 7:** The association between Cov-Anx and negative religious coping for each religious identity

Religious identity	Mean Cov-Anx (sd)	Mean NRC (sd)	*β*_*NRC*_	SE	*t*-value	Fit (*R*^2^)
Religious	1.72 (1.25)	2.54 (0.90)	0.72	0.05	21.73	0.53
Spiritual	1.55 (1.20)	2.32 (0.91)	0.79	0.06	18.59	0.62
Unsure	1.91 (1.03)	2.46 (0.71)	0.56	0.14	5.82	0.31
Agnostic	2.16 (1.13)	2.61 (0.86)	0.69	0.08	11.55	0.48
Atheist	1.55 (1.35)	2.06 (1.04)	0.85	0.08	13.50	0.72

^*^ *p* < .05. *Cov-Anx* Covid-19 anxiety, *NRC* negative religious coping

Another ANOVA was conducted to determine whether there were significant differences in levels of negative religious coping reported between religious identities as it held the strongest association with Cov-Anx scores in previous analyses collapsing across religious identity. A significant overall ANOVA *F*(4, 951) = 6.948, *p* < 0.001 was observed. Follow-up contrasts using Tukey’s HSD revealed that individuals identifying as atheist reported the lowest levels of NRC, with NRC also lower for individuals identifying as spiritual versus religious (all *p’s* < 0.05).

Because NRC was found to be most strongly associated with Cov-Anx in previous analyses, we looked to see the consistency of that relationship within each religious identity, especially given the individual group differences. A series of linear regressions for each religious identity were conducted using NRC as a predictor of Cov-Anx. These analyses revealed that the relationship between NRC and Cov-Anx was reliable within each religious identity (see Table 6), despite the group differences in NRC. In summary, regardless of one’s religious identification, NRC is positively associated with levels of Cov-Anx.

## COVID-19 Anxiety and COVID-19 Experience

Finally, a series of analyses were conducted to determine whether experiencing a COVID-19 diagnosis (through oneself, a close friend, or relative) would impact the relationship between religious coping, experiences, behaviors, and COVID-19 anxiety. First, a pair of independent samples t-tests were conducted. It was determined that there was no difference in Cov-Anx for those with (*M* = 1.76) or without (*M* = 1.74) COVID-19 experience (*t*(951 < 1.0)). Additionally, there was no difference in levels of negative religious coping for those with (*M* = 

2.48) or without (*M* = 2.43) COVID-19 experience (*t*(955) = 0.87, *p* = 0.39)), so no additional analyses were conducted.

## Discussion

The results of this study provide both theoretical and practical implications regarding the role of religious practice and religious coping during a global pandemic. We had predicted a negative association between reported levels of positive religious coping and reported levels of COVID-19 anxiety, potentially reflecting how individuals turn to such methods to alleviate significant stressors in their life during a pandemic. At best, this hypothesis was only partially supported in a regression analysis that also included negative religious coping and religious behaviors and beliefs, revealing a far more complicated picture about the role of religious coping in alleviating psychological distress.

This finding is not consistent with findings by Thomas and Barbato (2020), or Pirutinsky et al. (2020), who both found that positive religious coping served as a protective buffer against negative psychological outcomes in individuals reporting strong religious affiliation/religious identity and close ties to those communities (i.e., Muslims, and Orthodox Jews, respectively). While it is possible that cross-sectional data may fail to account for the tendency to use religion as a coping mechanism in times of stress, the hierarchical multiple regression (Table 5) showed that negative religious coping was more strongly associated with COVID-19 anxiety than positive religious coping. Therefore, while people may have turned toward religion to cope with COVID-19-related stress, the data suggest that the potential harm of negative religious coping may outweigh the potential benefits of positive religious coping.

Indeed, their findings are consistent with work indicating that positive religious coping can be more beneficial to people who identify as more religious (e.g., Pargament et al., 2001), as well as align well with research indicating that high levels of identification or affiliation with a specific social identity are related to increased levels of subjective well-being (DeRossett & Harvey, in prep; Kesler & Wann, 2020). Our findings regarding positive religious coping and COVID-19 anxiety are more consistent with work by Pargament et al. (2011) noting that positive religious coping is generally shown to be consistently associated with measures of positive psychological constructs and well-being (Pargament et al., 2011). In the current study only negative psychological constructs (i.e., Cov-Anx) were examined. Pargament et al. (2011) note that only occasionally is positive religious coping consistently inversely associated with negative constructs like anxiety, and our results support that argument.

We found stronger support for our hypothesis that negative religious coping is associated with higher levels of COVID-19 anxiety, and indeed our findings suggest that negative religious coping accounts for the greatest amount of the variance in levels of COVID-19-oriented anxiety. This held true regardless of the frequency with which individuals engaged in religious practices or behaviors, their overall experience with the sacred or divine, and their religious self-identity, and is consistent with the general literature examining the relationship between negative psychological constructs and religious coping (Pargament et al., 2011). Negative religious coping can reflect an ominous view of the world, and in the midst of a global pandemic, such a view makes sense. This becomes especially important as nationally, measures used to prevent the spread of COVID-19 included the closing of houses of worship, limiting people’s ability to turn to others for spiritual guidance, and connectedness in the same ways they could prior to social distancing and related limitations on gathering.

The relationships between COVID-19 anxiety and both religious experience and frequency are particularly interesting. In the hierarchical regression model (Table 5) religious behavior frequency was positively related to COVID-19 anxiety, while experience was negatively associated. Greater recent frequency of religious beliefs and behaviors was related to an increase in COVID-19 anxiety. This may be due to an overall increase in stress due to the COVID-19 pandemic which resulted in greater levels of prayer, meditation, or other religious behaviors to cope. It could also be due to higher anxiety attached to religious activities as they are often conducted in group settings which were unsafe during the COVID-19 pandemic.

Inversely, overall experience was negatively associated with COVID-19 anxiety. This aligns with concurrent research that found a positive relationship between affect and religion during the early stages of the COVID-19 pandemic (Zacher & Rudolph, 2021). Higher scores indicated a greater belief in God, prayer, and service attendance throughout one’s life. Holding a past foundation of religious beliefs and behaviors appeared to be related to less stress during the COVID-19 pandemic. However, the present study found that negative religious coping held a stronger association with Cov-Anx than positive religious coping. This suggests that while religion may act as a buffer for negative well-being outcomes, the manner of engagement with a religious identity is an important factor.

Interestingly, the relationship between negative religious coping and COVID-19 anxiety was significant regardless of religious identity. Greater levels of negative religious coping were associated with greater levels of COVID-19 anxiety in individuals who identified as agnostic, atheist, or unsure. These findings suggest that negative religious coping is harmful regardless of religious identity and should be avoided for individuals who seek to obtain the greatest levels of subjective well-being. Furthermore, individuals identifying as agnostic reported the greatest levels of NRC which calls for further research into the topic. Agnosticism is identified by high levels of skepticism and uncertainty regarding religion, and it seems that may be closely related to feelings of shame, guilt, or abandonment in times of stress. Future studies should investigate the relationship further.

## Study Limitations

An important limitation and consideration of this study is the fact that it was conducted in a single time period over a few days and relatively early (~ 6 months) into the course of the pandemic. A fuller understanding of the complexity of ways in which religious coping, both negative and positive, impact anxiety resulting from a pandemic, would be better gained through a longitudinal study, to be able to account for shifting dynamics of the pandemic itself. Our findings do, nonetheless, offer some insight into the relationship between religious coping and a newly identified form of anxiety that will likely remain prevalent until the threat of COVID-19 is significantly reduced. Additionally, the cross-sectional nature of the data does not allow for causal interpretation of our findings. Longitudinal data would be needed to assert causality.

### Implications

The data suggest that individuals should avoid engaging in NRC regardless of religious identity. This is in line with past research (Pargament et al., 2000) but the current study shows that the suggestion is still true during a global pandemic. PRC was not found to be an effective buffer against the stress and concern delivered via the early stages of the COVID-19 pandemic in the USA. The existence of negative coping behaviors seemed to outweigh the benefits provided by positive strategies.

The strength of NRC further highlights the importance of positive and effective clerical leadership during harmful events. As indicated by past research (e.g., CITE), religious leaders wield great levels of power and influence among their congregations. To engage in harmful coping strategies (i.e., the ills are a form of punishment or karma) may only result in skepticism, helplessness, and negative mental and physical well-being for those individuals. Therefore, leaders should seek to focus on positive and honest messaging. Religiosity has been found to be a buffer during the pandemic in other studies (e.g., Zacher & Rudolph, 2021), but harmful messaging and cognition only seems to worsen an already dark situation.

## Declarations

### Availability of Data and Material

Data and analyses are stored on OSF and are available at the following link (https://osf.io/j7t5f/) and https://doi.org/10.17605/OSF.IO/J7T5F.

### Code Availability

All code is available with the data and analyses on OSF.
